# Halofuginone — The Multifaceted Molecule

**DOI:** 10.3390/molecules20010573

**Published:** 2015-01-05

**Authors:** Mark Pines, Itai Spector

**Affiliations:** 1 The Volcani Center, Institute of Animal Science, P.O. Box 6, Bet Dagan 50250, Israel; E-Mail: itais222@gmail.com; 2 Department of Animal Sciences, Hebrew University of Jerusalem, Rehovot 76100, Israel

**Keywords:** malaria, fibrosis, inflammation, autoimmunity, Th-17, apoptosis

## Abstract

Halofuginone is an analog of febrifugine—an alkaloid originally isolated from the plant *Dichroa febrifuga*. During recent years, halofuginone has attracted much attention because of its wide range of beneficial biological activities, which encompass malaria, cancer, and fibrosis-related and autoimmune diseases. At present two modes of halofuginone actions have been described: (1) Inhibition of Smad3 phosphorylation downstream of the TGFβ signaling pathway results in inhibition of fibroblasts-to-myofibroblasts transition and fibrosis. (2) Inhibition of prolyl-tRNA synthetase (ProRS) activity in the blood stage of malaria and inhibition of Th17 cell differentiation thereby inhibiting inflammation and the autoimmune reaction by activation of the amino acid starvation and integrated stress responses. This review deals with the history and origin of this natural product, its synthesis, its known modes of action, and it’s various biological activities in pre-clinical animal models and in humans.

## 1. Introduction

Identification of bioactive small molecules and confirmation of their activity is crucial for both academic research and pharmaceutical applications. The plant kingdom provides a reservoir of natural products that play highly significant roles in drug discovery and development. Thus, biodiversity represents an unlimited source of novel chemical entities that offer promise as novel, efficacious and safe therapies for various diseases [1]. There is a current perception that bioactive compounds are obtained through computer-modeling, bioinformatics, chemical genetics, high-throughput screening, and other drug-discovery methods [2]. However, the history of science contains many cases in which serendipity prevailed where other approaches failed, and halofuginone—an analog of febrifugine, an alkaloid originally isolated from the plant *Dichroa febrifuga*—is an excellent example of a bioactive molecule discovered by serendipity. In this review we will describe the history and origin of this natural product, its synthesis, its known modes of action, and it’s various biological activities in preclinical animal models and in humans.

## 2. The Origin of Halofuginone and Its Synthesis

In China, *Dichroa febrifuga* Lour., which belongs to the Saxifragaceae family has been used for centuries against malarial fever. Extracts from either the roots or the leaves were effective for treating chicks infected with *Plasmodium gallinaceum* and in clinical cases of malaria [3]. Among 600 plant extracts tested for their antimalarial effects, the extract of *D. febrifuga* was found to be one of the most effective [4]. An active quinazoline-type alkaloid isolated from the roots and having the molecular structure C_16_H_21_0_3_N_3_ was designated dichroin B [5], and later renamed febrifugine [6]. The alkaloids febrifugine and its stereoisomer, isofebrifugine, have been identified as the active components of the plant, and both exhibited *in vitro* antimalarial activity against chloroquine-sensitive and chloroquine-resistant *P. falciparum* [6,7]. Febrifugine was found to be effective against *P. vivax* [8] and more active than quinine against *P. lophurae*, *P. gallinaceum*, and *P. cynomolgi* [9]. Because the high antimalarial activity was accompanied by gastrointestinal toxicity associated with, e.g., diarrhea, vomiting, and liver toxicity [10,11,12], the structure of febrifugine was used as a lead compound in the synthesis of some active molecules with lower toxicity [13] that inhibited parasite growth both *in vitro* [14] and *in vivo* [15]. In addition, synthesis of a series of febrifugine derivatives as antimalarial drugs was performed by structural modifications at the quinazoline ring, the linker, or the piperidine ring [16,17], and antimalarial drug-discovery models were developed for screening and predicting efficacious febrifugine analogs [18]. It has been demonstrated that most febrifugine analogs bearing a modified or unmodified 4- quinazolinone moiety are active, but analogs produced through modification of the side chain attached to the N_3_ position of the 4-quinazolinone ring have proved to be ineffective. Furthermore, a synthetically prepared racemic febrifugine was reported to be about half as effective as natural febrifugine. These results suggested that the 4-quinazolinone moiety, the nitrogen atom of the piperidine ring, and the hydroxyl group are necessary for the antimalarial activity, and that the absolute configuration of these functional groups plays an important role [19].

**Figure 1:**
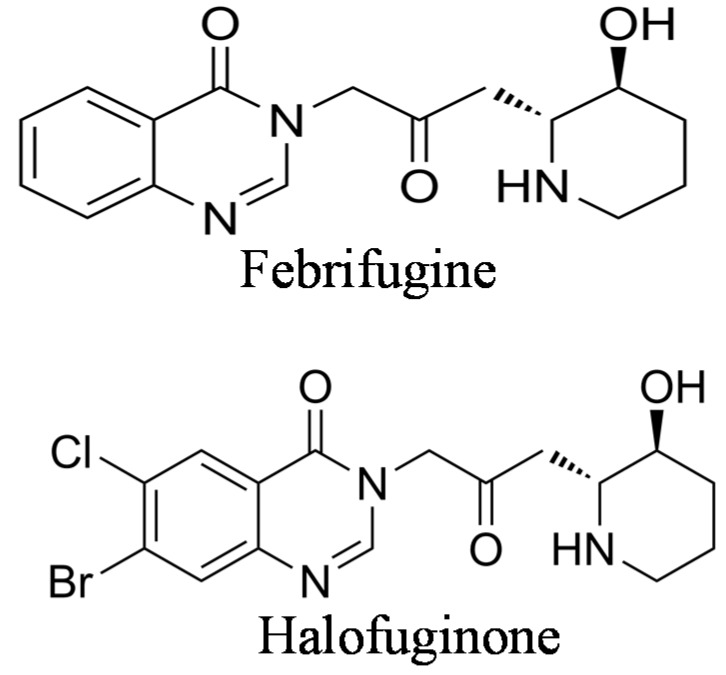
Chemical structures of febrifugine and halofuginone.

Halofuginone {7-bromo-6-chloro-3-[3-(3-hydroxy-2-piperidinyl)-2-oxopropyl]-4(3*H*)-quinazolinone} (Figure 1) is one of the febrifugine analogs used worldwide in commercial poultry production [20,21]. Halofuginone hydrobromide is an FDA–approved feed additive for prevention of coccidiosis in broiler chickens and growing turkeys [22]; also, other halofuginone salts are used against protozoan parasites in cattle [23]. As in febrifugine, the piperidine ring was found to be essential for halofuginone activity [24]. Because of the high interest in febrifugine and its analogs, including halofuginone, as antimalarial drugs, many reports on their synthesis were published during the years ([19,25,26,27,28], to cite just a few). Some of the early synthesis procedures encountered various problems because of uncertainty regarding the absolute stereochemistry. It was found that under certain conditions febrifugine and isofebrifugine could interconvert [29]. Recently, an elegant review was published that described the chemical complexity of the molecules involved and the problems encountered during the synthesis of febrifugine, isofebrifugine and halofuginone [30].

## 3. Halofuginone as an Antimalaria Therapy

The malarial burden of mortality and morbidity continues to rise in several developing countries in Africa, South America, and Asia. Malaria-related deaths remain alarmingly high, partly because of emergence of drug-resistant strains of malaria parasites. Thus, there is a constant need for novel high-efficacy antimalarial therapies that affect various stages in the parasite life-cycle. Of a series of febrifugine analogs halofuginone was the most active against *P. falciparum* growth *in vitro*, and displayed curative effects in *P. berghei*-infected mice [14]. Chemicals with antimalarial effects showed differing patterns against ring stages, trophozoites, and schizonts of *P. falciparum* in culture, whereas halofuginone acted with equal speed on all 3 stages [31]. Halofuginone affects both the asymptomatic liver stage that is the first stage of the *Plasmodium* parasite’s life cycle as well as sporozoite propagation within liver cells [32] and the blood stage that elicits characteristic malaria symptoms [33]. In the liver stage, halofuginone inhibited the *P. berghei* sporozoite load in HepG2 cells, with an IC50 value of 17 nM, without affecting sporozoite traversal. Analysis of the structure/activity relationships indicated that the addition of bromide on the quinazoline ring preserved the antimalarial efficacy of halofuginone and lowered its cytotoxicity for the host cells. The antimalaria mode of action of febrifugine and its analogs probably involved interaction with prolyl-tRNA synthetase (ProRS) in the blood stage. Halofuginone was found to inhibit ProRS activity causing intracellular accumulation of uncharged tRNA and mimicking reduced cellular proline availability. This function requires ATP that directly locks onto and orients two parts of halofuginone onto human ProRS, so that one part of halofuginone mimics bound proline and the other mimics the 3' end of bound tRNA [34]. Halofuginone interacted strongly with the prolyl-tRNA synthetase of *P. falciparum*, which is resident exclusively in the parasite’s cytoplasm, within the asexual blood stage in a non-competitive binding mode. The antimalarial activity of synthesized febrifugine and halofuginone analogs was determined by using *in vitro* assays: against chloroquine-sensitive and -resistant *P. falciparum* strains for susceptibility; and against two mammalian cell lines—neuronal cell line NG108 and macrophage cell line J774—for cytotoxicity. The IC_50_s of halofuginone were observed to be the best among the synthesized derivatives of febrifugine [27].

## 4. Halofuginone as Antiprotozoan in Poultry and in Ruminants

Coccidiosis and cryptosporidiosis are parasitic diseases caused by protozoa; they develop in various regions in the gut affecting poultry flocks and cattle, respectively, and they cause mild to severe lesions, weight loss, diarrhea and increased mortality [35,36,37]. In poultry halofuginone was used as a bromide salt added to the diet; in ruminants it was administered orally as halofuginone lactate. In poultry halofuginone was effective against various *Eimeria* species [38]: it acted early in the life cycle of coccidia [39], was effective against infections of *Eimeria mitis* in young broiler chickens [40], inhibited the parasite’s invasion of the host’s cecum during early stages of the life cycle, and later disturbed the parasite’s development by vacuolation of the schizonts [21]. In calves, halofuginone was effective in reducing the risk of cryptosporidiosis [41,42] and decreased the intensity of diarrhea and fecal oocyst counts, when used in either preventive or therapeutic mode [43]. In lambs, halofuginone reduced diarrhea caused by cryptosporidiosis and reduced death rate [44]. The bromide salt of halofuginone was efficacious against *Cryptosporidium parvum* also *in vitro* [45].

The toxicity and the side effects of the racemic halofuginone limited the effective doses that could be used. In attempts to increase the therapeutic window for its application, trans-enantiomers (2*R*,3*S*)-(+) and (2*S*,3*R*)-(−) of halofuginone lactate were prepared [46]. The (2*R*,3*S*) enantiomer was more efficacious against *C. parvum in vitro*, and in mice its toxicity *in vivo* was higher than that of its optical antipode (2*S*,3*R*). These results suggested that the activity against *C. parvum* and the mammalian toxicity both reside within the same enantiomer, and that there is no advantage in using a specific enantiomer rather than the racemic halofuginone lactate.

## 5. Halofuginone as an Antifibrotic Agent

Fibrosis is the end result of chronic inflammatory reactions induced by a variety of stimuli; it leads to destruction of organ architecture and impairment of organ function and, if highly progressive, it eventually leads to organ malfunction and death. Fibrosis is characterized by high levels of extracellular (ECM) proteins, especially collagen type I. The key cellular mediator of fibrosis comprises the myofibroblasts, which, when activated, serve as the primary ECM-producing cells. Transforming growth factor β (TGFβ), the matrix metalloproteinases (MMPs), and the tissue inhibitor of metalloproteinases (TIMPs) play a crucial role in fine regulation of the ECM turnover, which is altered in most pathological states associated with fibrosis. The lack of specific inhibitor(s) of any component of the ECM in general, and of collagen type I in particular, limited the success of fibrosis treatment.

**Figure 2:**
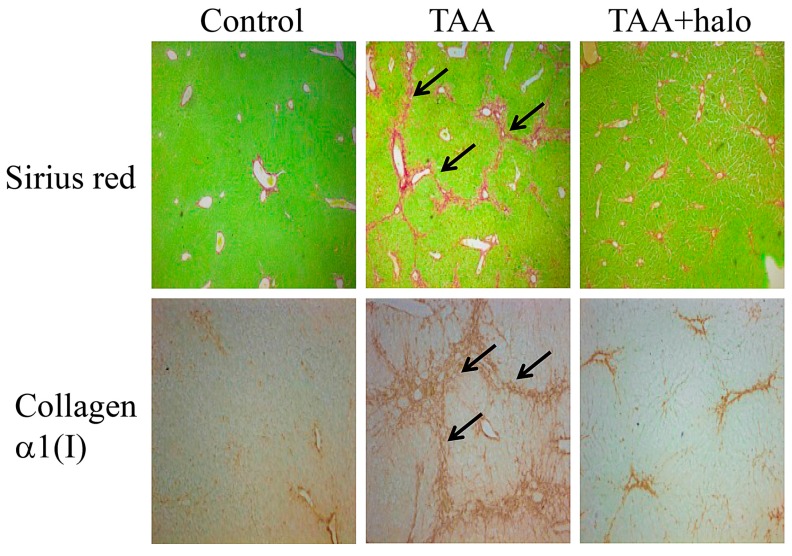
Histological analysis of liver sections. Liver biopsies were taken from mice treated with thioacetamide (TAA) to induce liver fibrosis (125 μg/kg), mice treated with halofuginone (7.5 μg/mouse) both injected intraperitoneal (IP) 3 times a week for 4 weeks, or in a combination of the two. The sections were stained with Sirius red for collagen (upper panel, stained red) and for collagen α1(I) gene expression by *in situ* hybridization (lower panel). In the control mice collagen and cells expressing collagen α1(I) gene were observed around the blood vessels. When treated with TAA the livers exhibited a marked increase in collagen content displaying bundles of collagen surrounding the lobules that resulted in large fibrous septa and increase in cells expressing the collagen α1(I) gene (arrows). Halofuginone treatment prevented the TAA-dependent increase in the collagen content and in the number of the cells expressing the collagen α1(I) gene

The antifibrotic properties of halofuginone were discovered by serendipity and were investigated in scores of preclinical animal models and in humans [47,48,49,50,51]. In culture, halofuginone attenuated collagen α1(I) gene expression by murine, avian and human skin fibroblasts derived from human corneal [52], scleroderma and chronic graft-*versu*s-host disease (cGvHD) patients [53]. In animal models in which excess of collagen is the hallmark of the disease, halofuginone prevented increase in collagen synthesis; these models included mice afflicted with cGvHD, and tight skin (Tsk) mice [54,55], rats with pulmonary fibrosis [56], rats that developed adhesions at various sites [57,58,59], and rats and mice with hepatic and pancreatic fibrosis (Figure 2 and [60,61,62]).

Most importantly, halofuginone can elicit resolution of established fibrosis—a capability that sets it apart from all other preventive antifibrotic agents. In rats with established hepatic fibrosis [63], and in the *mdx* mouse model of Duchenne Muscular Dystrophy (DMD) [64] halofuginone elicited reductions in the levels of collagen, collagen α1(I) gene expression, and α-smooth-muscle-positive cells, all of which are characteristic of the fibrotic condition, and this resulted in complete resolution of the fibrosis. Furthermore, regeneration of the liver, which was blocked in rats with established fibrosis, occurred at an almost normal rate in halofuginone-treated rats [65]. In the Tsk mice that developed cutaneous hyperplasia and connective tissue abnormalities halofuginone treatment caused a decrease in the pre-existing fibrotic condition, as indicated by changes in collagen gene expression, collagen content, and skin morphology [55].

**Figure 3:**
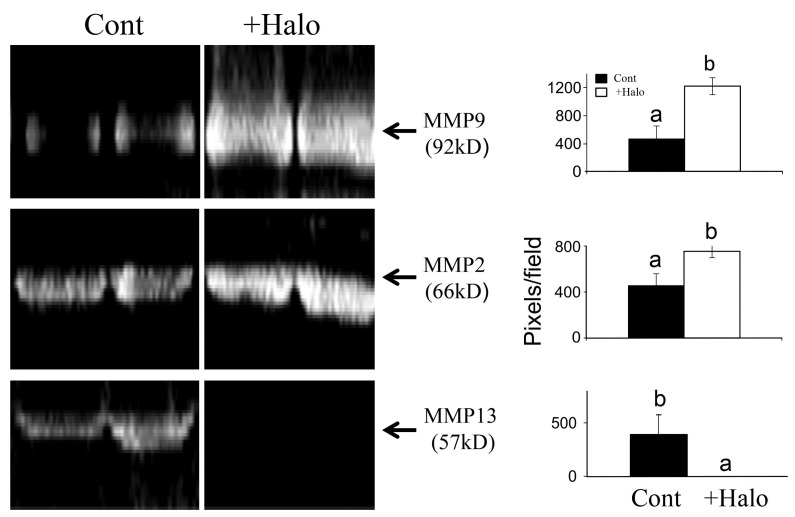
Effect of halofuginone on MMPs activity. Pancreatic tumors were established in Athymic-Nude (CD1 nu/nu) mice by implanting subcutaneously 10^6^ Panc2 human pancreatic tumor cells. Halofuginone was injected intraperitoneal (IP) 3 times a week starting one week after tumor cells implantation. Tumors (300 mg) were taken for MMPs activity analysis by zymography. Increase in MMP9 and MMP2 and decrease in MMP13 were observed in the tumors of halofuginone-treated mice compare to the untreated mice. Each column represents 15 sections of 5 different tumors and represents as the means ± SE according to Duncan’s multiple-range test. Columns with different letters differ significantly (*p* < 0.05).

The ability of halofuginone to elicit resolution of pre-existing fibrosis is probably due to its ability to reduce collagen synthesis and simultaneously to increase collagenase activity [66,67] by augmenting synthesis of the TIMPs [63] that regulate MMPs activity (Figure 3).

In most animal models of fibrosis, regardless of the tissue, halofuginone had a minimal effect on collagen content in the nonfibrotic animals, whereas it exhibited a profound inhibitory effect in the fibrotic organs. This suggests that the regulation of the low-level house-keeping expression of collagen type I genes differs from that of the over-expression induced by the fibrogenic stimulus, which is usually an aggressive and rapid process. TGFβ is one of the leading candidates for eliciting over-production of ECM proteins in various fibrotic conditions, and it inhibits the matrix-degrading enzymes [68]. The regulation of matrix proteins in general and of collagen type I gene expression in particular by TGFβ involves the Smad signaling pathway [69]. Halofuginone was found to overcome TGFβ-induced collagen synthesis in human [53] and Tsk [70] skin fibroblasts. In various fibrotic models no effect of halofuginone was observed on expression of the TGFβ receptor gene or on TGFβ levels [62,70,71]—a finding that supports the hypothesis that the halofuginone target is downstream in the TGF_β_ pathway. Halofuginone *in vitro* reduced Smad3 protein [52], inhibited TGFβ-dependent Smad3 phosphorylation and elevated expression of the inhibitory Smad7 in a variety of cell types, such as fibroblasts, hepatic (HSCs) and pancreatic (PSCs) stellate cells, tumor cells, and myoblasts [55,62,72,73,74]. In balloon-injured rat common carotid arteries halofuginone reduced collagen type 1—but not type 3 production—and effectively blocked Smad3 phosphorylation in smooth muscle cells, which is known to promote smooth muscle cell proliferation, migration, and intimal hyperplasia. Halofuginone is the one of the first reported small molecules that has favorable effects on all three major processes involved in restenosis [75]. In mouse models representing muscular dystrophies [76,77,78] and in various tumors [74,79] halofuginone inhibited Smad3 phosphorylation *in vivo*. The inhibition of Smad3 phosphorylation was due, at least in part, to halofuginone-dependent activation of Akt MAPK/ERK and p38 MAPK phosphorylation [73].

## 6. Halofuginone in Cancer, Angiogenesis, and Metastasis

The growth and spread of tumor cells depends on establishment of an adequate blood supply. Angiogenesis—the development and growth of blood vessels—has a pivotal role in tumor growth, progression, invasiveness, and metastasis.

**Figure 4:**
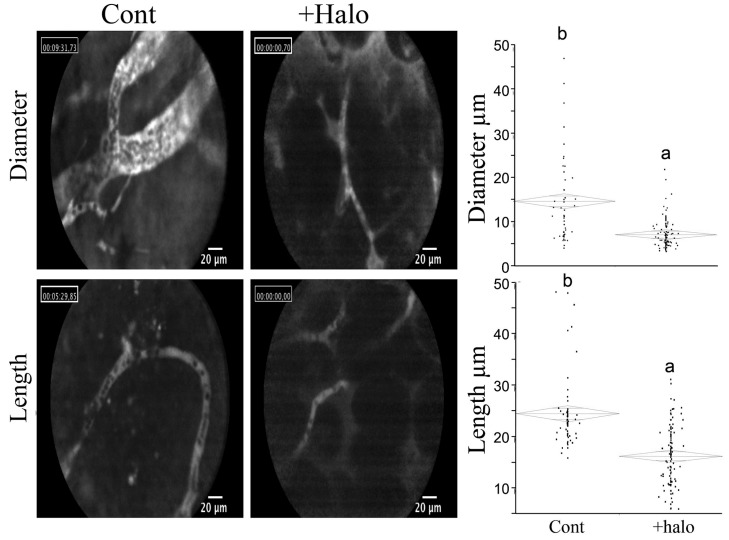
Effect of halofuginone on blood vessels within pancreatic tumor xenografts. Pancreatic tumors were established in Athymic-Nude (CD1 nu/nu) mice by implanting subcutaneously 10^6^ Panc2 human pancreatic tumor cells. Halofuginone was injected intraperitoneal (IP) 3 times a week starting one week after tumor cells implantation. When tumors volume reached 200 cm^3^, Fluorescein isothiocyanate-dextran (FITC-Dextran) was injected via the tail vein and blood vessels diameter and length were visualized by confocal microscopy. The results are of at least 70 blood vessels from different tumors. Columns with different letters differ significantly (*p* < 0.05). Note the decrease in both blood vessel diameter and length in the halofuginone-treated mice.

Halofuginone treatment resulted in profound inhibitory effects on representative sequential events in the angiogenic cascade, such as abrogation of endothelial cell MMP-2 expression, basement membrane invasion, capillary tube formation, and vascular sprouting, as well as deposition of sub-endothelial ECM *in vitro*. *In vivo* halofuginone inhibited neo-vascularization induced by basic fibroblast growth factor (bFGF) in a mouse corneal micropocket assay [80], and inhibited angiogenesis in glioma spheroids implanted in: nude mice [81], bladder carcinoma [82], Wilms tumor [83], cutaneous melanoma [84], pancreas cancer [85], and prostate cancer [86]. Moreover, halofuginone inhibits the diameter and length of blood vessels within the tumors (Figure 4).

In most of these cases inhibition of angiogenesis was accompanied by inhibition of the fibroblasts-to-myofibroblasts transition, reduction in tumor stroma ECM, and inhibition of tumor growth. Metastasis is the most fatal feature of malignant tumors; it accounted for more than 90% of tumor-related mortality, and distant-organ or tissue metastasis is a sign of poor prognosis in cancer patients. Successful metastasis requires not only a local niche to support primary tumor growth but also a metastatic niche consists of various ECM components and the enzymes that remodel them to enable disseminated cancer cells to survive colonize and proliferate at a distant site. In chemically induced, spontaneously metastasizing hepatocellular carcinoma [87] and in melanoma xenografts [88] halofuginone inhibited lung and bone metastasis, respectively.

Thanks to its unique modes of action halofuginone is a most suitable candidate for combination therapy. Halofuginone synergized with low doses of docetaxel in prostate cancer, with vincristine and dactinomycin in Wilms’ tumor, and with gemcitabine in pancreatic xenografts, which resulted in significant reductions in tumor volume and weight—comparable with the effects achieved by high doses of the respective chemotherapies [74,89].

## 7. Halofuginone and Apoptosis

Apoptosis is a latent built-in mechanism of regulated cell death, characterized morphologically by cell shrinkage, plasma membrane blebbing, nuclear condensation, and fragmentation of inter-nucleosomal DNA. The dead cell is packaged into membrane-bound apoptotic bodies, which are engulfed and removed by neighboring cells or tissue phagocytes [90].

With one exception, halofuginone induced apoptosis in all tested cell types. In benign tumors halofuginone inhibited cell proliferation of both leiomyoma and autologous myometrial cells in a dose-dependent manner, by inhibiting DNA synthesis and inducing apoptosis [91]. In acute promyelocytic leukemia cell lines the halofuginone-dependent apoptosis was associated with up-regulation of genes involved in cell cycle regulation [92]. Halofuginone induced apoptosis in multiple myeloma (MM) cells, even in the presence of growth factors, without inducing cytotoxicity. This was associated with depletion of mitochondrial membrane potential, and cleavage of poly(ADP-ribose) polymerase and caspases-3, 8, and 9, as well as down-regulation of anti-apoptotic proteins, including Mcl-1 and X-IAP [93]. Halofuginone inhibition of T-cell proliferation was correlated with an increase in cell apoptosis and a decrease in proline uptake, which suggests involvement of the amino acid starvation response (AAR) [94]. In pancreatic xenografts, halofuginone caused apoptosis of myofibroblasts that invaded the tumors [74], and increased apoptosis in human melanoma cells [88] by inhibiting Smad3 phosphorylation downstream of the TGFβ pathway. In human breast cancer cell lines, halofuginone induced apoptosis associated with inhibition of cell migration and of matrix-protein degradation [95].

The only case, so far, in which halofuginone reduced apoptosis was in Duchenne Muscular Dystrophy (DMD): in the *mdx* mouse model of DMD, halofuginone decreased apoptosis of the satellite cells that can re-enter the cell cycle to proliferate and differentiate into myoblasts, which results in increased myoblast survival. This was associated with reduction in the pro-apoptotic protein Bax and induction in the levels of the anti-apoptotic proteins Bcl2. It is interesting to note that increase in apoptosis was observed in the myofibroblasts invading the same dystrophic muscle [96]. Although more research is required for understanding what makes the satellite cell apoptosis in the dystrophic muscle unique in its response to halofuginone, this phenomenon makes halofuginone a useful drug that eliminates the unwanted myofibroblasts simultaneously with helping to mitigate undue cell loss.

## 8. Halofuginone Inflammation and Autoimmunity

T helper 17 (Th-17) cells, a distinct subset of CD4^+^ T cells with IL-17 as their major cytokine, orchestrate the pathogenesis of inflammation, and any dysregulated Th17 cells contribute to inflammatory and autoimmune diseases [97]. For example, IL-17 was over-expressed in inflamed lung endothelial cells in rheumatoid arthritis, and in serum samples from systemic lupus erythematosus patients. IL-17 is a pro-inflammatory cytokine which induces other cytokines, chemokines, and prostaglandins. Th17 cells secrete not only IL-17A, but also IL-17F, IL-21, IL-22 and IL-23; and these cytokines most likely cooperate to induce multiple inflammatory and hematopoietic effects on epithelial, endothelial, and fibroblastic cells [98].

Halofuginone selectively inhibited mouse and human Th17 differentiation *in vitro* by activating the AAR; it induced the AAR, and protected mice from Th17-associated experimental autoimmune encephalomyelitis *in vivo* [99]. This probably occurred by activation of the integrated stress response (IRS), which is a coping mechanism that supports cells experiencing metabolic, oxidative, and hypoxic stresses, and regulates amino acid metabolism and resistance to oxidative stress [24]. Global expression profile of halofuginone targets in epithelial cells reveals the ISR is activated by halofuginone in mammary epithelial cells. Halofuginone altered the expression of multiple genes, involved in a variety of biological processes including signal transduction, transcriptional regulation, redox and cell metabolic homeostasis demonstrating that halofuginone exerts broad transcriptional effects [24]. Halofuginone induced the expression of signature Atf4/Integrated Stress Response effector genes including Atf5, Ddit3, Trib3, Ndrg1, Gadd45a and Slc1a4. Ischemia reperfusion (IR) injury is an acute, multifactorial stress initiated by a temporary stoppage of blood flow that results in tissue damage, up-regulation of pro-inflammatory cytokines, chemokines, and cell surface adhesion molecules, and recruitment of innate immune cells. Halofuginone which activated the AAR by mimicking proline deprivation, protected against renal ischemic injury, resulting in reduced inflammation and preserved organ function. Halofuginone action required the amino acid sensor and eIF2α (eukaryotic translation initiation factor 2α) kinase Gcn2 (general control non-derepressible 2), implicating the AAR and translational control in stress protection [100]. In an animal model of vulvovaginal candidiasis, inhibition of Th17 differentiation led to significant inhibition of IL-17 production [101] and in mice model of estrogen-deficient osteoporosis halofuginone reduces the abundance of Th-17 cells and prevents estrogen-deficient osteoporosis by diminishing bone resorption without impacting osteogenesis [102]. The effects of halofuginone on Th17 differentiation involved increased signaling of extracellular signal-regulated kinase (ERK), and reduced expression of signal transducer and activator of transcription 3 (STAT3) and of nuclear factor of activated T cells cytoplasmic 1 (NFATc1) [103]. When knockout mice that lacked IL-17 expression in NKT-like cells were used as a model of acute GvHD, halofuginone inhibited Th17 differentiation and function, resulting in reduced disease severity [104].

## 9. Any Links between the Biological Activities of Halofuginone?

**Figure 5:**
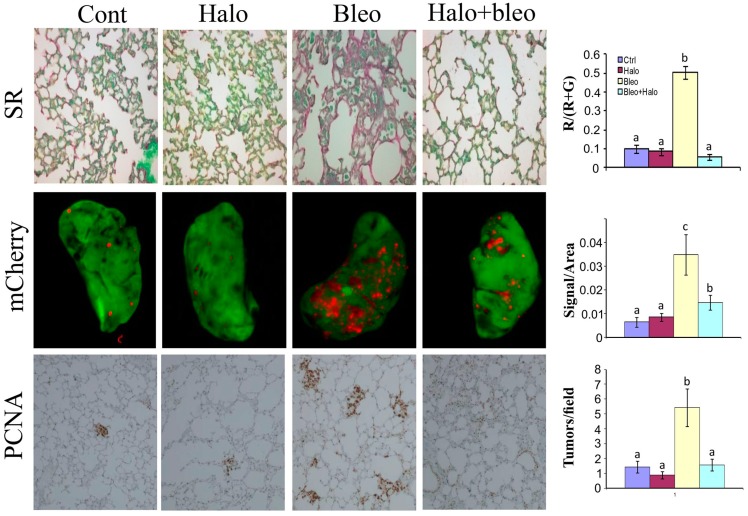
Effect of fibrosis on lung tumor development and progression. Lung fibrosis was induced by intraperitoneal daily injections of increasing concentrations of bleomycine to mice (25, 100, 200 300 and 400 μg/week). Note the increase in fibrosis in the bleomycine-treated mice (upper panel, stained red with Sirius red for collagen) compare to the untreated or to the bleomycine+halofuginone groups. After 5 weeks when lung fibrosis was established, 3 × 10^5^ B16F10 human melanoma tumor cells infected with mCherry (red) were injected via the tail vein. After additional 2 weeks, lung biopsies were immunostaining with proliferating cell nuclear antigen (PCNA) antibodies for cell proliferation (lower panel) and mCherry tumor were visualized by Maestro™ non-invasive fluorescence imaging system (middle panel). Each column represents at least 20 photos taken from different mice. Columns with different letters differ significantly (*p* < 0.05). Note that more tumors are established in the fibrotic tissue and halofuginone that reduced fibrosis reduced the number of tumors established in the lungs.

A major question that needs to be answered is whether all the described biological activities—and perhaps others, as yet unknown—of halofuginone should be attributed to a single mode of action or to multiple pathways. The two described modes of action known so far are inhibition of fibrosis and of fibroblasts-to myofibroblasts transition by inhibition of Smad3 phosphorylation down-stream of the TGFβ signaling pathway [50,51], and inhibition of Th17 differentiation, resulting in inhibition of the inflammation and autoimmune reaction by activation of the AAR response by binding to prolyl-tRNA synthetase [99,105,106]. The TGFβ pathway may represent at least one possible link between the two pathways. In mice Th17 differentiation requires initiation by TGFβ and IL-6, expansion by IL-21, and stabilization by IL-23 [107]. In humans, the combination of TGFβ and IL-21 was sufficient to induce differentiation in naïve T cells. There are at least two functional subclasses of Th17 cells, distinguished by their development in the presence or absence of TGFβ; also, some Th17 cells can produce their own TGFβ [108]. TGFβ [109] and latent TGFβ complexes on cell surfaces are involved in Th17 cell differentiation [110]. The effect of TGFβ on Th17 cell differentiation was concentration-dependent [111], and probably involved the TGFβ–Smad regulatory network [112]. Moreover, myofibroblasts isolated from gastric cancer induced Th17 cell differentiation when co-cultured with CD4^+^ T cells to a greater extent than normal myofibroblasts [113], and halofuginone prevented cutaneous GvHD [101] and concanavalin A-induced liver fibrosis by affecting Th17 cell differentiation [114] which suggests a direct link between the myofibroblasts/fibrosis pathway and the Th17 pro-inflammatory pathway, both of which are required for tumor development. In contrast, both TGFβ-independent and -dependent pathways of Th17 differentiation were described [115], and no TGFβ was required for induction of Th17-dependent experimental autoimmune encephalomyelitis [116]. The inflammation and fibrosis that follow infections with plasmodium [117,118], parasites [119], chronic active hepatitis B and C [120], and HIV-1 [121] destroyed the tissue microenvironment and impaired tissue/organ functions that then could no longer support the normal resident cell population, including CD4^+^ T cells. Halofuginone treatment that prevents the inflammation and fibrosis processes preserves the tissue architecture intact and enables it to combat the pathogenesis. Furthermore, tumor cells develop and metastasize more efficaciously in a fibrotic tissue [74,79], therefore any reduction in tissue fibrosis will reduce the risk of cancer (Figure 5).

## 10. Human Clinical Efficacy of Halofuginone

As a proof of principle, halofuginone was locally applied on the skin of a patient with cGvHD; evaluation of skin biopsies 3 months later revealed marked decreases in collagen α1(I) gene expression and collagen content, accompanied by functional improvement in neck rotation, without local or systemic toxicity, and no side effects were observed [122]. The antifibrotic effect of halofuginone was also demonstrated in AIDS-related Kaposi sarcoma [123]. In a scleroderma trial, local halofuginone treatment for 3 months caused a statistically significant reduction in the mean total skin score used to assess the severity of the disease [124].

Orally administered halofuginone was evaluated with favorable results, in a phase I clinical trial in patients with advanced solid tumors, thus demonstrating that therapeutically effective plasma levels can be reached safely. Patients were treated with escalating doses of halofuginone tablets at doses ranging from 0.5 to 3.5 mg/day. The “acute” maximum tolerated dose (MTD) was reached at 3.5 mg/day and the dose-limiting toxicities (DLT) were nausea, vomiting, and fatigue. No DLT was observed at 1 mg/day and the recommended dose for chronic administration was defined as 0.5 mg/day with the requirement of antiemetic to control DLTs [125]. At present, halofuginone in a slow-release formulation to avoid any DLTs is being evaluated in an FDA-approved Phase 1b open label, single- and multiple-ascending-dose study to evaluate its safety, tolerability, and pharmacokinetics in patients with DMD, in which fibrosis is the main complication.

## 11. Concluding Remarks

**Figure 6:**
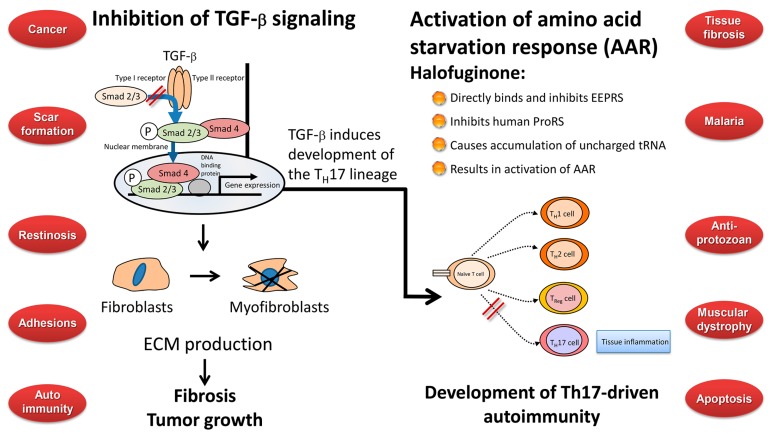
Halofuginone mechanism of action and targets. Halofuginone affects metabolic process such as the TGFβ and IL-17 signaling pathways that are involved in several biological activities encompassing a wide spectrum of endpoints. These include malaria cancer, fibrosis and more.

In recent years a lot of attention was focused on halofuginone and its analogs, in light of its wide range of beneficial biological activities against malaria, parasitic, and fibrosis-related diseases, and as an inhibitor of solid and plasma-cell cancers, and Th17-mediated inflammatory autoimmune diseases (Figure 6). Two pathways have been suggested for the biological activity of halofuginone which may be connected. In fibrosis and in cancer (left side) the TGFβ derived from the inflammatory cells binds to the constitutively active type II TGFβ receptor kinase that trans-phosphorylates the type I receptor. Activated type I receptor kinase then phosphorylates Smads 2/3, which subsequently recruit Smad4. The activated Smads 2/3–Smad 4 complex then translocates to the nucleus where it regulates specific gene expression. The TGFβ-dependent gene expression causes quiescent fibroblasts differentiation to myofibroblasts, which are responsible for the major increase in matrix synthesis in fibrosis and in tumor stroma which impedes normal function. Halofuginone inhibited TGFβ-dependent Smad3 phosphorylation, causes reduction in fibroblasts differentiation, reduction in the levels of ECM proteins and inhibition of fibrosis and tumor growth. Recently, a second mechanism has been suggested for the halofuginone-dependent inhibition of autoimmune diseases which involves selective prevention of the development of Th17 cells by activating the AAR—the amino acid starvation response (right side). Naïve CD4^+^ T cells differentiate into diverse effector and regulatory subsets to coordinate immunity to pathogens while establishing peripheral tolerance. Besides T_H_1 and Th2 effector subsets, naïve T cells can differentiate into proinflammatory T helper 17 (Th17) cells. These cells are key regulators of autoimmune inflammation. The mechanism by which halofuginone inhibits the AAR is by binding to glutamyl-prolyl-tRNA synthetase (EPRS) and inhibiting prolyl-tRNA synthetase activity (ProRS) causing intracellular accumulation of uncharged tRNA and mimicking reduced cellular proline availability. AAR activation selectively inhibits Th17 differentiation. Thus, halofuginone could potentially be used to address any autoimmune or inflammatory disease associated with Th17 cells. It should be noted (middle) that TGFβ is required for facilitation of differentiation of the inflammatory Th17 cell subset which suggests the existence of a link between the TGFβ and AAR pathways. The blue circles that are randomly aligned represent the various targets of halofuginone. Trying to correlate all the biological activities associated with a single molecule is a very complex task. On the one hand, protecting the tissue architecture and the specific niche by inhibiting fibrosis and inflammation may enable the tissue to defend itself against implantation of parasite, malaria, and cancer cells. Inhibition of the TGFβ signaling common to many, if not all, fibrotic reactions may account for the anti-fibrotic properties observed in a wide range of species, organs, tissues, and maladies. However, on the other hand, there are some discrepancies that do not fit these models: halofuginone’s effect of apoptosis, which is usually associated with minimal inflammation; the diversity in apoptosis in differing cell types; inhibition or activation of the NF-κB pathway in different cancer cells [66,126], the disparity in blocking Smad3 phosphorylation in smooth muscle cells and in balloon-injured rat carotid arteries [75], modulation of the expression of some ECM remodeling proteins without affecting TGFβ signaling [24]. Moreover, the effects of halofuginone on autoimmune disease models are cytoprotective, which suggests that enhancement of apoptosis or decrease of proliferation are not likely to be the molecular mechanism employed by halofuginone in this case [99].

It should be noted that halofuginone did not result from any drug discovery program, and its structure was not designed specifically for any of its biological activities. All the preclinical and clinical studies that involved halofuginone have been conducted on racemic material. Thus, a specifically designed chemistry approach that uses halofuginone as an advanced lead structure, together with *in vivo* biological assays will help to decipher its mode of action, on the one hand, and, on the other hand, will lead to a new and much needed arsenal of drug candidates that may be more specific with respect to the various biological actions, better tolerated, and more efficacious.
